# An exploratory cross-sectional study of the effects of ongoing relationships with accompanying patients on cancer care experience, self-efficacy, and psychological distress

**DOI:** 10.1186/s12885-023-10856-9

**Published:** 2023-04-22

**Authors:** Marie-Pascale Pomey, Monica Iliescu Nelea, Louise Normandin, Cécile Vialaron, Karine Bouchard, Marie-Andrée Côté, Maria Alejandra Rodriguez Duarte, Djahanchah Philip Ghadiri, Israël Fortin, Danielle Charpentier, Mélanie Lavoie-Tremblay, Nicolas Fernandez, Antoine Boivin, Michel Dorval, Mado Desforges, Catherine Régis, Isabelle Ganache, Lynda Bélanger, Zeev Rosberger, Michel Alain Danino, Jean-François Pelletier, Thi Trinh Thuc Vu, Michèle de Guise

**Affiliations:** 1 Innovation Axis, Research Center of the Centre Hospitalier de L’Université de Montréal, (CHUM), Montréal, QC Canada; 2 Department of Health Management, Evaluation, and Policy, School of Public Health, Université de Montreal, 7101 Av du Parc 3E Étage, Montréal, QC H3N 1X9 Canada; 3 Research Chair in Evaluation of State-of-the-Art Technologies and Methods, Montréal, QC Canada; 4 Center of Excellence On Patient Partnership and the Public, Montréal, QC Canada; 5 Centre Hospitalier Universitaire - CHU de Québec- Université Laval, Québec, QC Canada; 6 HEC Montréal, Department of Management, Montréal, QC Canada; 7 Centre Intégré Universitaire de Santé Et Services Sociaux de L’Est-de-L’Île-de-Montréal, Hôpital Maisonneuve-Rosemont, Montréal, QC Canada; 8 Centre Hospitalier de L’Université de Montréal, Montréal, QC Canada; 9 Faculty of Nursing, Université de Montréal, Montréal, QC Canada; 10 Institut Universitaire en Santé Mentale de Montréal, Montréal, QC Canada; 11 Department of Family and Emergency Medicine, Faculty of Medicine, Université de Montréal, Montréal, QC Canada; 12 Université Laval – Faculty of Pharmacy, Québec, QC Canada; 13 Centre de Recherche du CHU de Québec - Université Laval, Québec, QC Canada; 14 Centre de Recherche du CISSS Chaudière Appalaches, Lévis, QC Canada; 15 Université de Montréal – Faculty of Law, Montréal, QC Canada; 16 Institut National d’excellence en Santé Et Services Sociaux (INESSS), Montréal, QC Canada; 17 Gerald Bronfman Department of Oncology, Lady Davis Institute for Medical Research, Jewish General Hospital &, McGill University, Montréal, QC Canada; 18 Centre Intégré de Santé Et de Services Sociaux de La Montérégie-Ouest, St-Hubert, QC Canada; 19 Yale Program for Recovery & Community Health, New Haven, CT USA

**Keywords:** Breast cancer, Accompanying patient, Peer support, Patient advisors, Patient care experience, Patient partnership, oncology

## Abstract

**Background:**

Centre hospitalier de l’Université de Montréal in Canada introduced accompanying patients (APs) into the breast cancer care trajectory. APs are patients who have been treated for breast cancer and have been integrated into the clinical team to expand the services offered to people affected by cancer. This study describes the profiles of the people who received the support and explores whether one-offs vs ongoing encounters with APs influence their experience of care, on self-efficacy in coping with cancer, and on their level of psychological distress.

**Methods:**

An exploratory cross-sectional study was carried out among patients to compare patients who had one encounter with an AP (G1) with those who had had several encounters (G2). Five questionnaires were administered on socio-demographic characteristics, care pathway, evaluation of the support experience, self-efficacy in coping with cancer, and level of psychological distress. Logbooks, completed by the APs, determined the number of encounters. Linear regression models were used to evaluate the associations between the number of encounters, patient characteristics, care pathway, number of topics discussed, self-efficacy measures in coping with cancer, and level of psychological distress.

**Results:**

*Between April 2020 and December 2021, 60% of 535 patients who were offered support from an AP accepted. Of these,* one hundred and twenty-four patients participated in the study. *The study aimed to recruit* a minimum of 70 patients with the expectation of obtaining at least 50 participants, assuming a response rate of 70%. There were no differences between G1 and G2 in terms of sociodemographic data and care pathways. Statistical differences were found between G1 and G2 for impacts on and the return to daily life (*p* = 0.000), the return to the work and impacts on professional life (*p* = 0.044), announcement of a diagnosis to family and friends (*p* = 0.033), and strategies for living with treatment under the best conditions (*p* = 0.000). Significant differences were found on the topics of cancer (*p* = 0.000), genetic testing (*p* = 0.023), therapeutic options (*p* = 0.000), fatigue following treatment (*p* = 0.005), pain and discomfort after treatment or surgery (*p* = 0.000), potential emotions and their management (*p* = 0.000) and the decision-making processes (*p* = 0.011). A significant relationship was found between the two groups for patients’ ability to cope with cancer (*p* = 0.038), and their level of psychological distress at different stages of the care pathway (*p* = 0.024).

**Conclusions:**

This study shows differences between one-time and ongoing support for cancer patients. It highlights the potential for APs to help patients develop self-efficacy and cope with the challenges of cancer treatment.

## Background

According to the World Health Organization (WHO), the global cancer burden in 2020 was estimated to be 18.1 million cases. Out of these, 9.3 million cases were diagnosed in men and 8.8 million in women. The most commonly diagnosed cancer in women was breast cancer, with 2,261,419 new cases reported in 2020, accounting for 12.5% of all cancer cases [1]. In the province of Quebec, as elsewhere in Canada and in other industrialized countries, cancer is the leading cause of death [2, 3]. In 2022, 58,400 Quebecers will be diagnosed with cancer, or 160 new cases per day. This number has been on the rise for several years and will increase in the coming years due to delays in screening and diagnosis as a result of the pandemic [4]. Among these cancers, breast cancer is the most common form of cancer in women: 6,970 women in Quebec are affected each year and 1,360 die from it [2].

In this context, the government set up the Programme québécois de cancérologie (PQC), which proposes an action plan aimed at improving the survival rate among people affected by cancer, their quality of life, and their access to efficient health care and services, that is adapted to user needs in the short, medium, and long term [5, 6]. Among the objectives pursued by this plan is improved emotional support for patients struggling with cancer [7]. However, a study has shown that, among the various dimensions of the care experience evaluated, provision of emotional support under the program was the least successful [7].

Regarding the quality of life mentioned above, it is well known that breast cancer and its treatment can have a profound effect on the quality of life of patients. These impacts, known as breast cancer sequelae, can range from physical symptoms such as pain and fatigue to psychological symptoms like anxiety and depression. These sequelae can have long-lasting effects and can greatly impact a patient's daily activities, work, and relationships. It is essential for healthcare providers to acknowledge and address the impact of breast cancer sequelae on their patients' quality of life to improve patient outcomes and overall well-being. The ultimate goal is to reduce the treatments that may negatively affect the quality of life of breast cancer survivors. This requires not only the efforts of physicians, but also attention and support from society, communities, social media, and institutions [8].

To support cancer patients in coping with physical and psychological distress, Takano et al. [9] developed a self-help workbook. The workbook is intended to improve communication with medical staff and improve quality of life. Moreover, in an editorial, Invernizzi et al. [10] explore quality of life in breast cancer patients and survivors. Breast cancer can have a significant impact on the quality of life of patients, but with effective screening programs and treatment protocols, the number of deaths from this disease has declined, and healthcare providers now aim to not only prolong patients' lives, but also improve their well-being during and after treatment. A precision medicine approach is necessary to address quality of life issues and improve decision making and treatment compliance in the journey of survivorship, with the goal of returning to the quality of life before the diagnosis or improving on it [11].

In line with the desire to improve the quality of life of breast cancer patients and offer personalized care, we propose here to introduce accompanying patients (APs) who follow the patient and who, in addition to being involved at the organizational level in their health care institution, are also trained to get involved in the support of patients throughout their care trajectory. APs are patients who have previously experienced an episode of cancer. They provide their experiential knowledge of living with the disease and the use of health services to patients recently diagnosed with this disease. APs complete the care offering and are considered full members of the clinical team [12]. They provide emotional, educational, informational, navigational, and empowerment support to patients who are going through an episode of cancer by mobilizing their own experience [12]. In this way, they may help cancer patients regain control over their health condition, engage in their care, and improve their care experience [13, 14].

Since April 2020, in the context of COVID-19, the Centre hospitalier de l’Université de Montréal has introduced APs into the care trajectory of patients affected by breast cancer. This coincided with the onset of the COVID-19 pandemic, which increased the need for emotional support [4, 15]. Patients experienced increased delays in accessing surgery or other cancer treatments [4], reduced contact times with healthcare professionals, shorter hospitalizations, and a prohibition on relatives accompanying patients to their medical appointments.

To assess the contribution made by APs, a research project, called PAROLE-Onco (Le Patient Accompagnateur, une Ressource Organisationnelle comme Levier pour une Expérience patient améliorée en oncologie / The Accompanying Patient, an Organizational Resource as a Lever for an enhanced oncology patient Experience), was proposed and funded.

The objectives of this study are to describe the profiles of the people diagnosed with breast cancer who received the support (at least one encounter with the AP) and to explore whether one-offs vs ongoing encounter with APs has an effect on their experience of care (the topics addressed, added value, satisfaction, delivery methods), on self-efficacy in coping with cancer, and on their level of psychological distress.

## Methods

### Study design and population

An exploratory cross-sectional quantitative study was conducted to compare patients who had had one encounter with an AP (G1) with those who had had several (G2) [12].

The study population includes all breast cancer patients affected by cancer followed at the Centre hospitalier de l’Université de Montréal who had participated in at least one encounter with an AP in the period from April 2020 to December 2021. Patients could receive the support at any time in their care pathway, from when they receive the diagnosis to the end of the treatment.

We aimed to recruit a minimum of 70 patients with the expectation of obtaining at least 50 participants, assuming a response rate of 70%. This was based on a previously established response rate of 70% [15, 16].

### Research materials

Five questionnaires (Q) were used for this study. The first (Q1) covers socio-demographic characteristics, the second (Q2) deals with the patient’s care pathway, and the third (Q3) focuses on the support experience, in which the patient could comment on the added value of encounters with an AP, the benefits/appreciation of the support, and the desired improvements. These three questionnaires were developed specifically for this study and were co-constructed with APs [7]. The other two questionnaires are validated questionnaires: The Communication and Attitudinal Self-Efficacy scale for cancer (CASE-cancer) (Q4) [16], and Kessler’s Psychological Distress Scale (K6) (Q5) [17, 18]. Authorization for their use was obtained from the authors. The CASE-Cancer questionnaire measures the ability to cope with cancer. This questionnaire includes: 3 dimensions (understand and participate in the care; maintain a positive attitude and seek and obtain information), 12 items and a 4-point Likert scale (1 = strongly disagree and 4 = strongly agree). As for the Kessler’s Psychological Distress Scale (K6) questionnaire on emotional states, it includes 6 items (In the past 30 days: 1) how often did you feel…nervous?; 2) how often did you feel…hopeless?; 3) how often did you feel…restless or not holding still?; 4) how often did you feel… so depressed that nothing could make you smile?; 5) how often did you feel…that everything was an effort?; 6) how often did you feel…worthless?) and a 5-point Likert scale (1 = Always and 5 = Never). The characteristics of each of the five questionnaires sent to the patients and the logbook completed by the APs are presented in more detail in a previous article [12].

The logbooks were also co-developed with the APs for the study. They were used to calculate the number of encounters per patient.

### Recruitment of patients to participate in the PAROLE-Onco research project

The inclusion criteria were as follows: 1) an understanding of written and spoken French: participants had to understand and speak French in order to understand the information provided and communicate effectively with the research team; 2) a diagnosis of or treatment for cancer: participants had to have already received a diagnosis of or treatment for cancer as an important condition for their participation in the project; 3) minimum age of 18 years: participants had to be at least 18 years old to participate in the project; 4) preliminary meeting with an AP: participants had to have had a preliminary meeting with an AP to discuss project details and ensure that their participation would be consistent with their wishes and needs. Patients with conditions such as a mental disability or severe chronic illness were accompanied, if the patient wished, but the research project was not presented to them.

The patients were recruited for the study by the APs themselves. At the end of the first encounter, the AP notified the patient of the PAROLE-Onco research project and invited her to participate. In some cases, the project was presented during subsequent encounters so as not to interfere with the support. If the patient expressed interest in participating, the AP forwarded the patient's email address to the coordinator. The coordinator then contacted the patient to explain the research to her, answer her questions and send her, by email, the information and consent form along with the electronic questionnaires.

The link to the Q1-5 questionnaires was sent a few days after the first meeting with the AP. A new link to Q3-5 was sent about 4 weeks later. In both cases, two reminders were sent, one and two weeks after the first email [19].

### Measures and variables

For the survey data, REDCap (Research Electronic Data Capture) was used, an application for building and managing online surveys and databases used to administer questionnaires, organize data collection, and extract and analyze data [20]. REDCap complies with the applicable data privacy laws in its role as a data processor of customer data, as indicated on the company’s website [20].

In addition, the CASE-cancer (Q4) and the K6 (Q5) scores were calculated in accordance with the literature [16-18], knowing that for Q4, the higher the score, the better the patient can cope with her cancer [16]. For Q5, the higher the score, the higher the patient's level of psychological distress [18].

The number of encounters was calculated from the logbooks by determining the number of encounters held before completing the Q4 and Q5 questionnaires.

### Sample construction

Since the objective was to assess the relation of counseling on self-efficacy in coping with cancer and on the level of psychological distress, data was included from patients who had had at least one encounter before completing the questionnaires (Q1 to Q5).

When data was missing for one of the questions used to calculate CASE-cancer or K6 scores, a value was imputed, based on the mean of the values of the patient's other responses [21].

### Statistical analysis

Given the asymmetrical distribution of the number of counseling sessions (most patients (65%) had only one encounter), a binary variable was constructed to identify the patients of G1 and G2 in order to measure the relationship between the experience of care, self-efficacy in coping with cancer, and level of psychological distress by group (G1 and G2).

Descriptive analyses are presented of the socio-demographic characteristics, the care pathway, the evaluations of the encounters, self-efficacy in coping with cancer, and the level of psychological distress by group (G1-G2). Descriptive analyses were also performed using Fisher's exact test to identify a significant relationship between two categorical variables [22]. For continuous variables, the difference in scores (for Q4) between G1 and G2 was analyzed using the Wilcoxon test [23].

Two linear regression models were estimated to assess the relationship between the number of encounters on self-efficacy in coping with cancer (CASE-cancer) and on the level of psychological distress (K6). For the first model, the dependent variable was the CASE-cancer score and for the second, it was the K6 score. The socio-demographic characteristics represented the independent variable, while the care pathway and the topics addressed in the encounters were co-variables.

Throughout the data analysis, the exact Fisher test and Wilcoxon test were used to report statistically significant findings, with the level of significance set at 0.05.

All the statistics were calculated using RStudio Version 1.4.1103 [24].

## Results

From April 2020 to December 2021, health professionals offered 535 patients the opportunity to receive support from an AP, and 321 (60.0%) accepted the offer. Among these patients, 197 (36.8%) agreed to participate in the PAROLE-Onco research project and 124 (23.2%) completed questionnaires Q1 to Q3 ((G1: 81 (65.3%) and G2: 43 (34.7%)). A total of 118 patients (22.1%) completed questionnaires Q4 and Q5, including 75 in G1 (63.5%), and 43 in G2 (36.4%).

### Selection of participants and implementation of support from APs

**Fig. 1:**
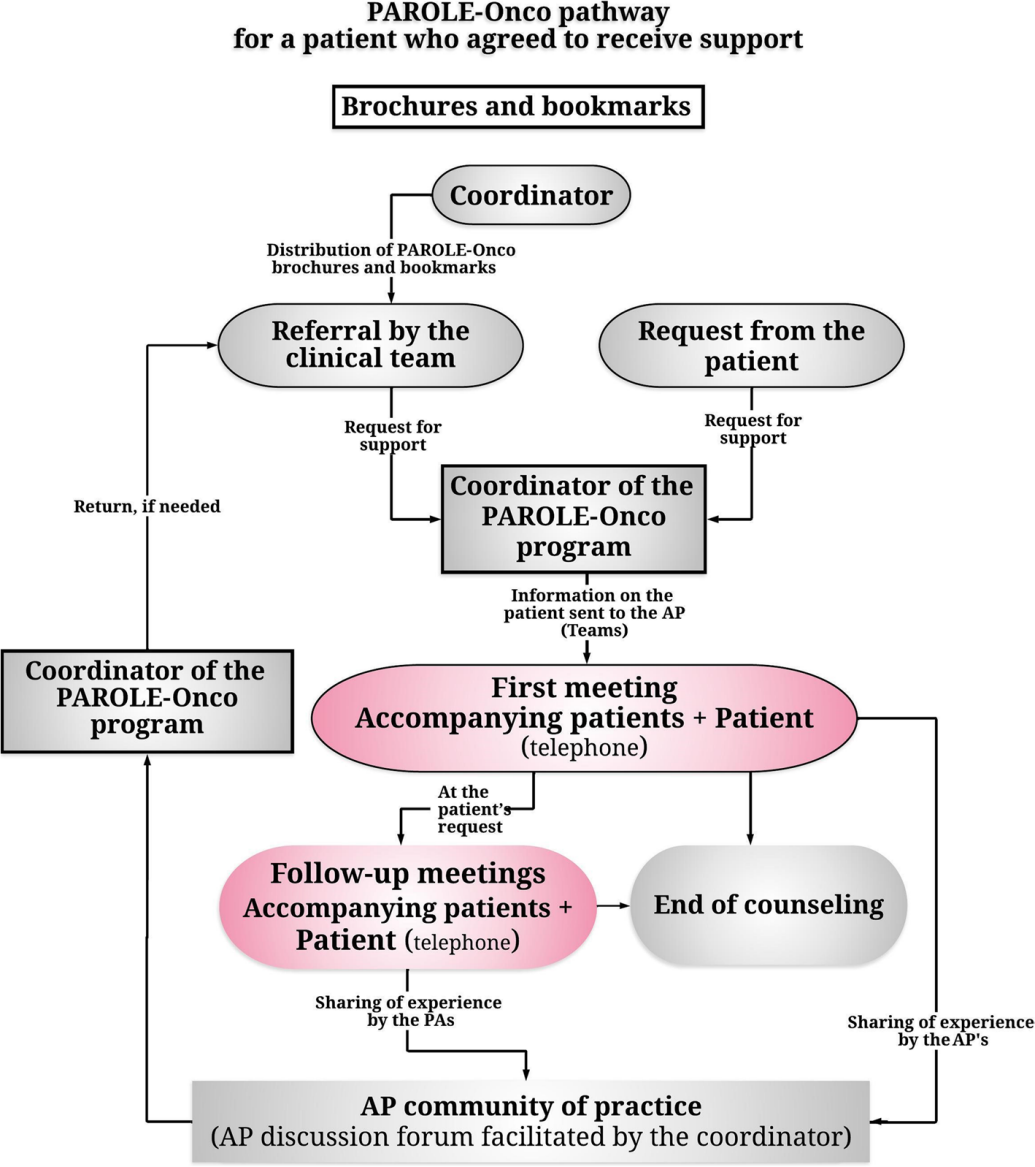
Process of accompanying a breast cancer patient through an accompanying patient

During a consultation with a patient affected by breast cancer, the health professionals (surgeons, hematologist-oncologists, radiation oncologists, nurses, psychologists, and social workers) would propose that the patient meet with one of the seven APs (Table 1). These APs had themselves been treated for breast cancer at the same university hospital (CHUM) and were subsequently recruited and trained, on a voluntary basis, to intervene with patients throughout the care trajectory and through all stages of the disease. If the patient accepted the support, relevant information (age, family situation, language of conversation, position in the care trajectory and treatment plan, if available) was transmitted by the program coordinator to the seven APs, anonymously, by secure messaging. Based on this information, one of the APs would indicate her interest in providing the patient with support, and the patient's personal details were sent to her, again by secure messaging. The AP would then contact the patient by telephone. During a period of support, the patient could request several encounters. In such cases, the patient would contact the program coordinator who followed up with the AP. In this way, the AP’s contact information would remain confidential. After each meeting, the AP would make an entry in a logbook that was added to the patient's medical file. If the AP thought that her discussions with the patient would be important for patient follow up by the clinical team, the AP would contact the coordinator, who sent the information, in a secure and timely manner, to the clinical team. The whole process is shown in Fig. 1.

**Table 1:** Characteristics of accompanying patients who have had a breast cancer trajectory

AP record	Age group	Family situation	Level of education	Clinical picture
AP 1	45–54 years	Person living alone	University degree	Metastatic cancer Hormone therapy Radiotherapy Partial surgery Reconstructive surgery Immunotherapy
AP 2	55–64 years	Couple with no children at home	High school diploma	BRCA genetic mutation DIEP (deep inferior epigastric perforators) Oophorectomy
AP 3	55–64 years	Couple with child/children at home	University degree	Oral chemotherapy Hormone therapy Radiotherapy Partial surgery Reconstructive surgery
AP 4	65–74 years	Couple with no children at home	University degree	Radiotherapy Complete surgery
AP 5	55–64 years	Couple with no children at home	University degree	Metastatic cancer Oral and intravenous chemotherapy
AP 6	65–74 years	Person living alone	University degree	Hormone therapy Radiotherapy Partial surgery
AP 7	25–34 years	Family with other persons	University degree	BRCA genetic mutation Oral chemotherapy Complete surgery Radiotherapy Reconstructive surgery

### Characteristics of the support

The 124 supported patients benefited from 224 meetings with an AP ((G1: 81 (36.1%) and G2: 143 (63.8%)). The average number of encounters per patient is 2.3, and the median is 1.5. A total of 30 patients in G1 (38.0%) and 20 in G2 (46.5%) met with an AP before the start of their treatment, and 41 patients in G1 (51.9%) and 22 (51.2%) in G2 met with an AP during treatment. Only 8 (10.1%) patients in G1 and 1 patient (2.3%) in G2 met with an AP at the end of their treatment. Thirty-three patients (26.6%) benefited from more than one encounter before agreeing to participate in the research project.

### Socio-demographic characteristics (Q1)

The socio-demographic characteristics of the participants show that most were born in Canada, were over 45 years of age, were in relationships, and were receiving help from their partner for their medical follow-up. They were also most often university graduates and had worked full-time in the previous 12 months. The analyses do not show any statistically significant difference between the patients in G1 and those in G2 (Table 2).

**Table 2:** Relationship between sociodemographic characteristics and patient’s care experience, with CASE-cancer and level of psychological distress

	**Total**	**G1**	**G2**		**CASE-cancer**		**Psychological distress**	
	**n (%)**	**n (%)**	**n (%)**	***p*** **-value**	**Mean**	***p*** **-value**	**Mean**	***p*** **-value**
**Age group**	*n* = 124	*n* = 81	*n* = 43	0.1857		0.355		0.183
25 – 34 years	11 (8.9)	10 (12.3)	1 (2.3)		36.90		9.90	
35 – 44 years	20 (16.1)	15 (18.5)	5 (11.6)		38.89		6.39	
45 – 54 years	36 (29.0)	24 (29.6)	12 (27.9)		37.32		7.77	
55 – 64 years	33 (26.6)	20 (24.7)	13 (30.2)		39.61		5.97	
65 years or older	24 (19.3)	12 (14.8)	12 (28.0)		39.59		6.13	
**Place of birth**	*n* = 124	*n* = 81	*n* = 43	0.5887		0.245		0.467
Canada	107 (86.3)	71 (87.6)	36 (83.7)		38.87		7.08	
Outside Canada	17 (13.7)	10 (12.3)	7 (16.3)		36.89		6.06	
**Composition of the household**	*n* = 123	*n* = 81	*n* = 42	0.5284		0.002^a^		0.105
Person living alone	40 (32.5)	24 (29.6)	16 (38.1)		40.47		6.46	
Couple without children at home	27 (22.0)	19 (23.5)	8 (19.0)		41.00		5.80	
Couple with child/children at home	35 (28.5)	26 (32.1)	9 (21.4)		36.00		7.18	
Single-parent family	14 (11.4)	8 (9.9)	6 (14.3)		36.00		9.07	
Family with other persons	1 (0.8)	0 (0.0)	1 (2.4)		36.00		6.17	
**Informal caregiver for medical follow-up**	*n* = 124	*n* = 72	*n* = 40	0.5611		0.316		0.632
Spouse	62 (55.4)	42 (58.3)	20 (50.0)					
Child	30 (26.8)	19 (26.4)	11 (27.5)					
Parent	31 (27.7)	15 (20.8)	16 (40.0)					
Friend	46 (41.1)	27 (37.5)	19 (47.5)					
Other	15 (13.4)	8 (11.1)	7 (17.5)					
Yes					38.45		6.99	
No					39.89		6.36	
**Highest level of education completed**	*n* = 124	*n* = 81	*n* = 43	0.6272		0.705		0.678
**Main occupation during the last 12 months**	*n* = 123	*n* = 81	*n* = 42	0.2822		0.372		0.886
Full-time worker	67 (54.5)	46 (56.8)	21 (50.0)		38.53		7.11	
Part-time worker	16 (13.0)	10 (12.3)	6 (14.3)		39.24		6.25	
Retired	22 (17.9)	11 (13.6)	11 (26.2)		40.10		6.55	
Other	18 (14.6)	14 (17.3)	4 (9.5)		36.89		7.29	
**Financial situation**	*n* = 124	*n* = 81	*n* = 43	0.5797		0.010^a^		0.121
Financially comfortable	47 (37.9)	13 (16.0)	17 (39.5)		40.34		6.27	
Sufficient income to meet basic needs	70 (56.5)	14 (17.3)	25 (58.1)		37.83		7.03	
Poor	7 (5.6)	19 (23.5)	1 (2.3)		34.33		10.67	
**Cancer**	*n* = 122	*n* = 79	*n* = 43	0.074		0.066		0.914
First diagnosis of cancer	108 (88.5)	67 (84.8)	41 (95.3)		38.66		6.95	
Cancer relapse	12 (9.8)	11 (13.9)	1 (2.3)		36.55		6.46	
Second or third cancer	2 (1.6)	1 (1.3)	1 (2.3)		46.00		7.00	
**Metastatic cancer**	*n* = 115	*n* = 74	*n* = 41	0.106		0.071		0.112
Yes	18 (15.7)	15 (20.3)	3 (7.3)		36.53		8.18	
No	97 (84.3)	59 (79.7)	38 (92.7)		39.14		6.52	
**Time to cancer diagnosis**	*n* = 122	*n* = 79	*n* = 43	0.724		0.334		0.203
Less than 1 month	19 (15.6)	12 (15.2)	7 (16.3)		38.95		7.90	
1 to 6 months	71 (58.2)	44 (55.7)	27 (62.8)		38.58		7.00	
7 to 11 months	19 (15.6)	12 (15.2)	7 (16.3)		37.44		5.89	
1 to 3 years	9 (7.4)	8 (10.1)	1 (2.3)		38.11		7.25	
More than 3 years	3 (2.4)	2 (2.6)	1 (2.3)		44.67		3.67	
**Stage of the care pathway**	*n* = 122	*n* = 79	*n* = 43	0.273		0.226		0.024^a^
Before treatment begins	50 (41.0)	30 (38.0)	20 (46.5)		37.6		7.88	
During treatment	63 (51.6)	41 (51.9)	22 (51.2)		39.54		5.97	
After treatment completed	9 (7.4)	8 (10.1)	1 (2.3)		37.62		7.88	
**Type of treatment or surgery received**	*n* = 122	*n* = 81	*n* = 43	0.538				
Intravenous chemotherapy	36 (29.5)	24 (29.6)	12 (27.9)					
Oral chemotherapy—swallowing tablets	8 (6.6)	8 (9.9)	0 (0.0)					
Hormone therapy	20 (16.4)	16 (19.8)	4 (9.3)					
Radiotherapy or brachytherapy	20 (16.4)	16 (19.8)	4 (9.3)					
Partial mastectomy	24 (19.7)	17 (21.0)	7 (16.3)					
Total mastectomy	23 (18.9)	15 (18.5)	8 (18.6)					
Reconstructive surgery	21 (17.2)	14 (17.3)	7 (16.3)					
Transplantation or grafting	1 (0.8)	0 (0.0)	1 (2.3)					
Medication for symptoms	18 (14.8)	13 (16.0)	5 (11.6)					
Alternative medicine	6 (4.9)	5 (6.2)	1 (2.3)					
Other	4 (3.3)	2 (2.5	2 (4.7)					
Don't know	0 (0.0)	0 (0.0)	0 (0.0)					
**Initiation of treatment**	*n* = 71	*n* = 48	*n* = 23	1.000		0.115		0.396
Less than 1 month	14 (19.7)	9 (18.8)	5 (21.7)		38.95		7.31	
1 to 6 months	34 (47.9)	23 (47.9)	11 (47.8)		38.58		5.27	
7 to 11 months	14 (19.7)	9 (18.8)	5 (21.7)		37.44		7.54	
1 to 3 years	5 (7.0)	4 (8.3)	1 (4.3)		38.11		8.25	
Over 3 years	4 (5.6)	3 (6.3)	1 (4.3)		44.67		4.33	

### Care pathway (Q2)

The patients in G1 and G2 do not have significantly different characteristics related to their care pathways, whether in terms of cancer diagnosis and stage, diagnostic delays, stage of care pathway, type of treatment and time of treatment initiation (Table 2).

### Support experience (Q3)

#### Topics addressed during the encounters with APs

The topics addressed during the support were grouped according to three main themes: (1) the impacts of cancer on daily life, (2) clinical issues, and (3) the organizational features of the provision of care (Table 3).

**Table 3:** Topics of discussion with APs during the encounters

Themes	Topics	Total	G1	G2	*p*-value
		*n* = 124 (%)	*n* = 81 (%)	*n* = 43 (%)	
**Impacts on daily life**	Impacts on and the return to daily life	57 (46.0)	20 (24.7)	27 (62.8)	0.000^a^
	Impacts on the children	16 (12.9)	9 (11.1)	7 (16.3)	0.414
	Impacts on marital and sexual life	5 (4.0)	2 (2.5)	3 (7.0)	0.340
	The return to work and impacts on professional life	20 (16.1)	9 (11.1)	11 (25.6)	0.044^a^
	Impacts on spiritual life	1 (0.8)	1 (1.2)	0 (0.0)	1.000
	How to announce a diagnosis to loved ones and social perceptions	24 (19.4)	11 (13.6)	13 (30.2)	0.033^a^
	Impacts on one’s finances and insurance	4 (3.2)	1 (1.2)	3 (7.0)	0.120
	Strategies for living with treatments in the best possible conditions	22 (17.7)	6 (7.4)	16 (37.2)	0.000^a^
	How to regain control over the disease	13 (10.5)	6 (7.4)	7 (16.3)	0.136
	Other information	2 (1.6)	0 (0.0)	2 (4.7)	0.118
**Clinical issues**	Announcement by the physician of the diagnosis of cancer or a genetic predisposition	25 (20.2)	13 (16.0)	12 (27.9)	0.158
	Cancer	51 (41.1)	22 (27.2)	29 (67.4)	0.000^a^
	Genetic testing	12 (9.7)	4 (4.9)	8 (18.6)	0.023^a^
	Therapeutic, surgical and reconstructive options for cancer and for breast prostheses	41 (33.1)	13 (16.0)	28 (65.1)	0.000^a^
	Ways to reduce risk in carriers of a genetic mutation that increases the risk of cancer	0 (0.0)	0 (0.0)	0 (0.0)	N/A
	Hormonal and reproductive issues, urinary dysfunctions	10 (8.1)	5 (6.2)	5 (11.6)	0.314
	Fatigue following treatment, impact on physical appearance and self-esteem	42 (33.9)	20 (24.7)	22 (51.2)	0.005^a^
	Pain and discomfort after treatment or surgery	38 (30.6)	15 (18.5)	23 (53.5)	0.000^a^
	Potential emotions and their management	62 (50.0)	29 (35.8)	33 (76.7)	0.000^a^
	Decision-making processes	17 (13.7)	6 (7.4)	11 (25.6)	0.011^a^
	Other information	1 (0.8)	0 (0.0)	1 (2.3)	0.347
**Organizational features**	One’s role as an accompanying patient and the PAROLE-Onco project	62 (50.0)	34 (42.0)	28 (65.1)	0.023^a^
	Roles played by various healthcare professionals	32 (25.8)	10 (12.3)	22 (51.2)	0.000^a^
	Roles played by external and internal organizations	20 (16.1)	10 (12.3)	10 (23.3)	0.130
	The care trajectory	46 (37.1)	17 (21.0)	29 (67.4)	0.000^a^
	Patients’ rights (e.g., refusing treatment, asking questions)	28 (22.6)	9 (11.1)	19 (44.2)	0.000^a^
	Where and how to get to medical appointments	8 (6.5)	4 (4.9)	4 (9.3)	0.447
	Financial support for patients and assistance with transportation	1 (0.8)	1 (1.2)	0 (0.0)	1.000
	Other information	10 (8.1)	5 (6.2)	5 (11.6)	0.314

^a^ Statistically significant findings

For the *impacts of cancer on daily life*, the results show statistically significant differences between the number of sessions and the themes of the impacts on daily life and the return to daily life (*p* = 0.000), the return to work and the impacts on professional life (*p* = 0.044), how to announce a diagnosis to loved ones and social perceptions (*p* = 0.033), and strategies for receiving treatment under the best conditions (*p* = 0.000).

At the level of *clinical issues*, statistically significant differences were found between the two groups for the topics of cancer (*p* = 0.000); genetic tests (*p* = 0.023); therapeutic, surgical, and reconstructive options in the case of cancer and breast implants (*p* = 0.000); fatigue following treatments, repercussions on physical appearance and self-esteem (*p* = 0.005); pain and discomfort after treatment or surgery (*p* = 0.000); potential emotions and their management (*p* = 0.000); and decision-making processes (*p* = 0.011).

As for the *organizational features*, among the 7 sub-themes, 4 were significantly different between G1 and G2 (the role as accompanying patient and the PAROLE-Onco project (*p* = 0.023); the role of the various health professionals (*p* = 0.000); the care trajectory (*p* = 0.000); and rights as a patient (*p* = 0.000).

#### Contribution made by the APs

We have classified the contributions made by APs into five categories: informational support, support for being a partner in one’s care, emotional support, educational support, and support on navigating the health system to be a partner in one’s care.

More than half of the participants reported that the AP’s contribution was centered on educational support (helping to live with one's illness), emotional support (validation of emotions), and informational support (transfer and validation of information). A significantly higher proportion of patients in G2 identified the APs’ contributions to be in all five categories, and especially in emotional support (G1 = 43.2%; G2 = 86.0%). The least identified category was support for being a partner in one's care (having a new perspective on decision making, participating and, above all, becoming a partner in one's own care) (G1 = 28.4%; G2 = 55.8%) (Table 4).

**Table 4:** Contribution made by APs

Contribution of support (AP’s contribution)	Total	G1	G2	*p*-value
	*n* **= 124** **(%)**	*n* **= 81** **(%)**	*n* **= 43** **(%)**	
Informational support: transfer and validation of information	67 (54.0)	34 (42.0)	33 (76.7)	0.000^a^
Support for being a partner in one’s care: communication/trust/decision making/etc	47 (37.9)	23 (28.4)	24 (55.8)	0.004^a^
Emotional support: validation of emotions	72 (58.1)	35 (43.2)	37 (86.0)	0.000^a^
Educational support: assistance with living with their disease	73 (58.9)	37 (45.7)	36 (83.7)	0.000^a^
Navigation support: help in using the health care system well and understanding who does what	57 (46.0)	27 (33.3)	30 (69.8)	0.000^a^

^a^Statistically significant findings

Table 5 presents the patients’ comments on the support they received. In terms of the added value of the support, they especially highlighted its contribution to reducing anxiety. Regarding potential improvements, the patients suggested ensuring that the care pathways between patients and APs are as similar as possible.

**Table 5:** Patients’ comments following the support (from comments on the Q3 questionnaire)

Value added by support	Benefits and appreciation of the support	Desired improvements
“There should be accompanying patients for all diseases! It would be beneficial for both the patients and the care teams.” (ID 284) “I really enjoyed talking with my AP. She gave me a different perspective. And the fact that she is about my age.” (ID 255) “It's these meetings that help me lower my anxiety and understand my journey better. I always feel much better afterwards.” (ID 301) “This help is invaluable, especially because it is the only help I’ve been offered. After being diagnosed with cancer, one lives with a deep fear of dying. You have to adjust to a mutilated body. We see our loved ones suffer, and the public health system doesn’t offer any psychological help […]. So, without an accompanying patient, I’d be alone with all my fears and anxieties, with no-one to talk to and put things in perspective.” (ID 353) “I needed to talk with people who are facing similar issues, besides the disease (young, single, childless). The accompanying patient helped me find resources to meet this need. […]” (ID 249)	“I only spoke with my AP once, but that was enough for me. I was very touched by her capacity to listen and by her involvement. I felt well that she understood my concerns, and then reassured. Thank you!” (ID 318) “Just one thing. Thank you for everything you do!” (ID 257) “The service was highly appreciated.” (ID 295) “The accompanying patient was particularly empathetic and caring. I am very grateful to have had access to her services.” (ID 348) “I would recommend that all new patients have this type of support. It’s so beneficial.” (ID 355) “I received a call from an accompanying patient, but I don't think I need it for the rest of the process, as I have my own way of dealing with it.” (ID 253)	“I would have liked to have had help at the beginning of the diagnosis rather than after the operation.” (ID 214) “I would have liked to have had an accompanying patient who would have been in the same situation as me, that is, someone living alone […]” (ID 290) “The general idea of the project is interesting. However, as far as I’m concerned, the conversation took place at a time when I still didn’t have all the data […].” (ID 221) “Later on, I would have liked to talk with a woman who had a mutation like me, who had been through something more similar to my experience.” (ID 221)

#### Evaluation of the experience in relation to the support

**Fig. 2:**
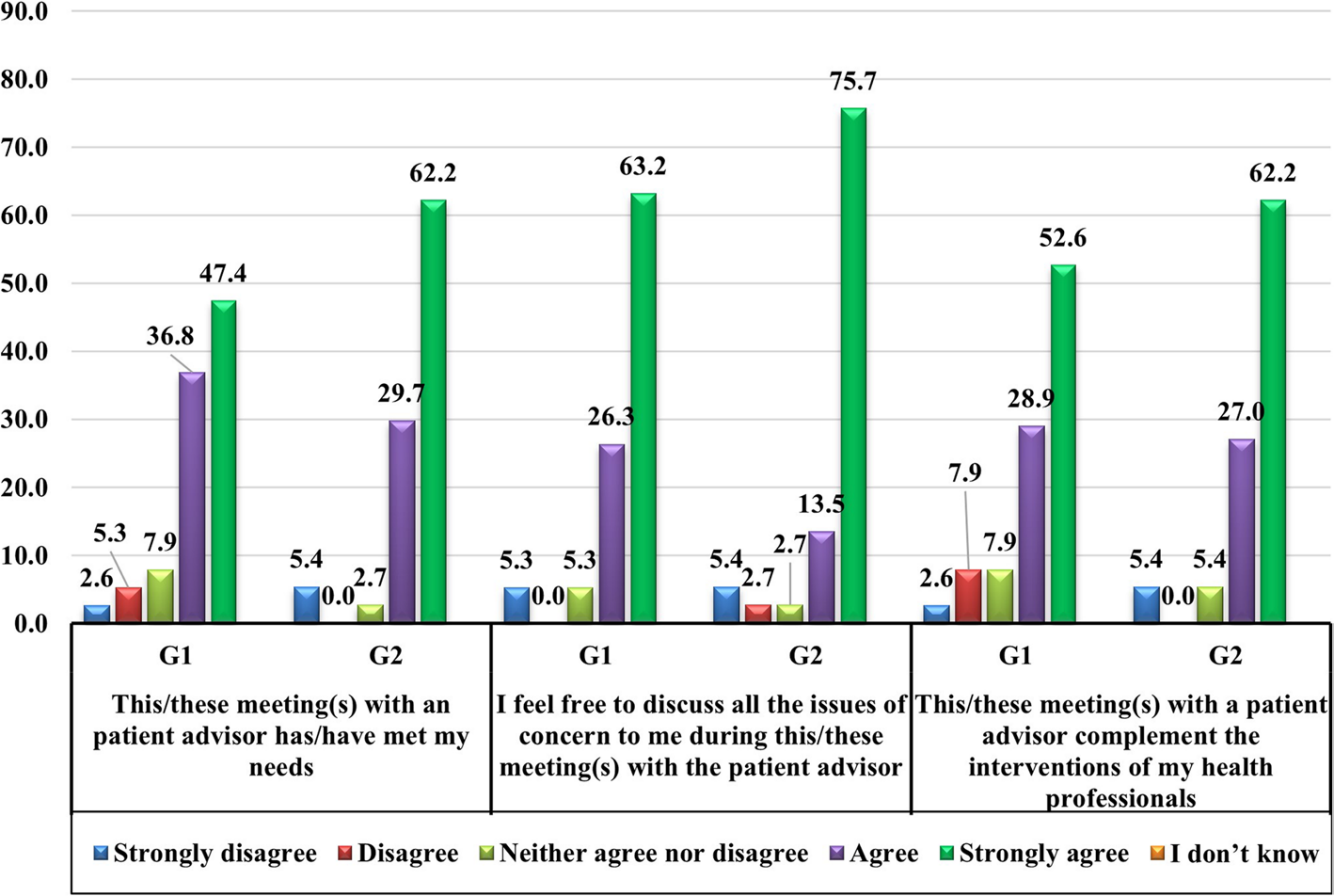
Assessment of the experience of patients following one (G1) or more (G2) encounters, in %

Figure 2 presents the evaluation of the patients’ experience following one or more encounters with an AP. In both groups, the patients reported that the encounters with an AP met their needs, that they felt free to discuss all the subjects of concern to them, and that these encounters complemented the interventions of their healthcare professionals. In addition, the patients in G1 (86.8%) and G2 (97.3%) are in complete agreement with deploying this support on a larger scale to help more patients with other tumor sites. There is no significant difference between G1 and G2.

#### Evaluation of delivery methods

**Fig. 3:**
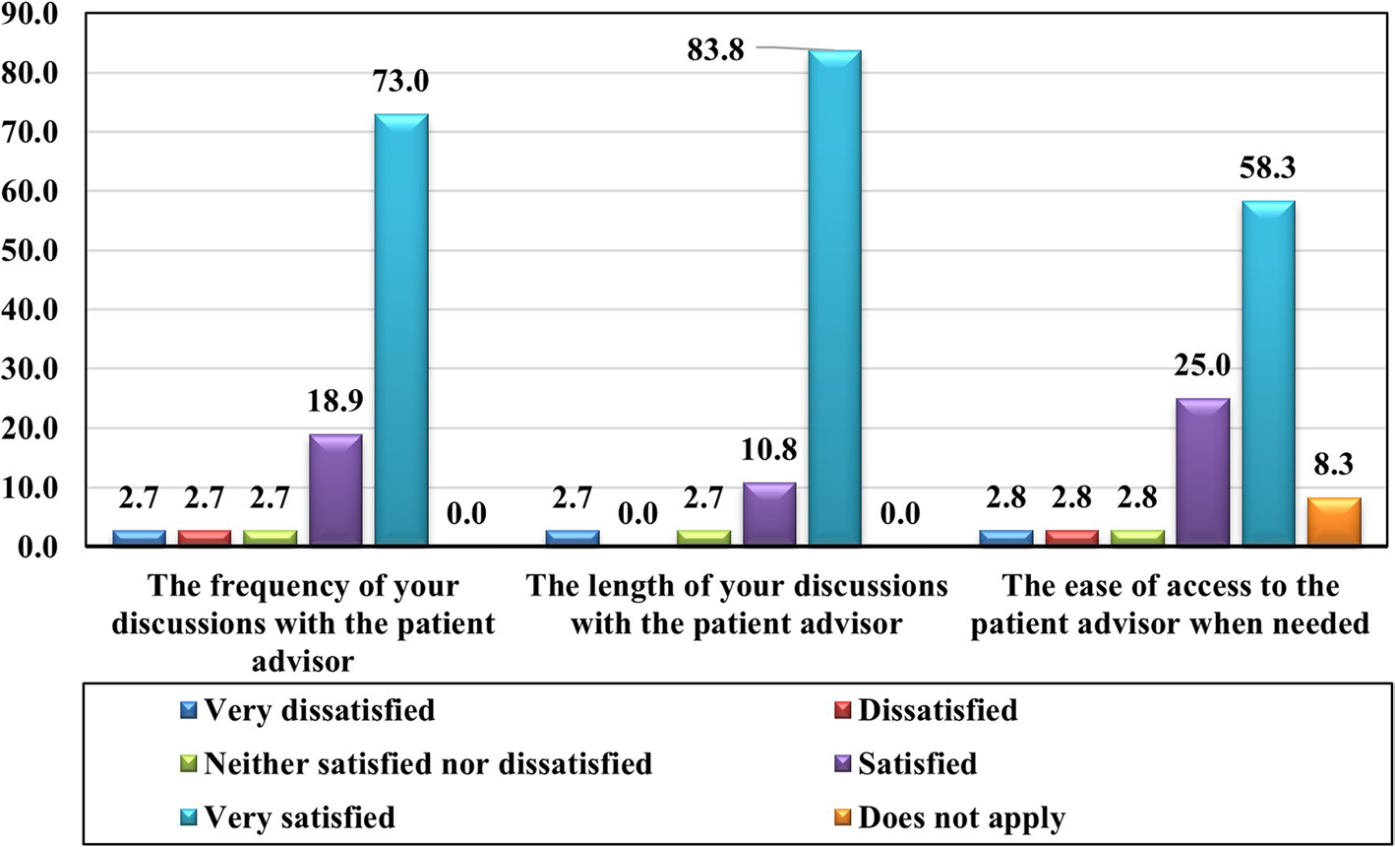
Evaluation of encounters by patients who met with their APs several times (G2), in %

Since this does not apply to patients who had only one encounter, the evaluation of the delivery methods (frequency, duration, and access) was only analyzed for the patients in G2 (Fig. 3). More than 90% of G2 patients reported that the frequency and duration of discussions with the AP was satisfactory. For 83.3% of patients, their ease of access to the APs was satisfactory.

#### Evaluation of support benefits

**Fig. 4:**
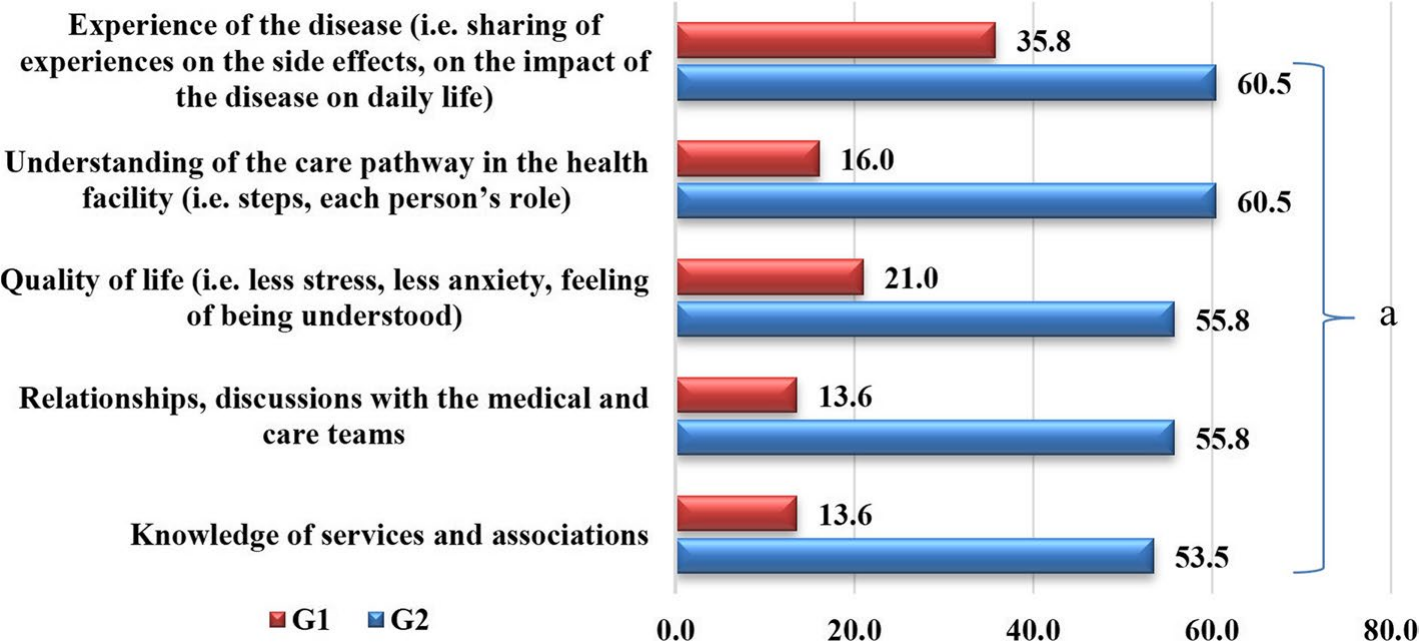
Evaluation of the benefits of support by G1 and G2 patients. ^a^Statistically significant findings

Figure 4 presents the proportion of patients per group (G1, G2) who perceived benefits from the support in different areas. A significantly higher proportion of G2 patients noted a benefit from the encounters in the areas of living with the disease (G1 = 35.8%; G2 = 60.5, *p* = 0.008), understanding of the care pathway within the health facility (G1 = 16.0%; G2 = 60.5, *p* = 0.000), quality of life (G1 = 21.0%; G2 = 55.8, *p* = 0.000), relationships, communication with medical and healthcare teams (G1 = 13.6%; G2 = 55.8, *p* = 0.000), and knowledge of services and associations (G1 = 13.6%; G2 = 53.5, *p* = 0.000).

### Communication and Attitudinal Self-Efficacy scale for cancer (CASE-cancer) (Q4)

The average CASE-cancer score of the patients in the study is 38.6, which is high, since the potential range of scores for this questionnaire is from 12 to 48. Looking more specifically at the average CASE-cancer score by socio-demographic characteristics and care pathway, we find a statistically significant difference for composition of the household (*p* = 0.002). Patients who live with a partner with a child at home, who are single parents, or who live with other people have a lower average CASE-cancer score and are therefore less able to cope with cancer than patients who live alone or with a partner without children at home. In addition, a statistically significant difference was found in patients who were financially comfortable compared to patients with financial concerns (*p* = 0.010) (Table 2).

Table 6 presents the frequency and mean CASE-cancer score according to the number of encounters for the three subscales: (1) understanding and participating in care, (2) maintaining a positive attitude, and (3) seeking and obtaining information. We found that G2 patients reported higher self-efficacy in the “understanding and participating in care” (60.5%), and “seeking and obtaining information” (51.2%) subscales. G2 patients reported significantly higher self-efficacy in the “understanding and participating in care” subscale (*p* = 0.038). For the “maintaining a positive attitude” subscale, a higher proportion of G1 patients reported low self-efficacy.

**Table 6:** Self-efficacy of patients with APs (*n* = 118)

CASE-cancer subscale	Support groups	CASE-cancer score^a^		Mean	Wilcoxon rank test	*p*-value
		**4 – 12 (low)**	**13 – 16 (high)**			
		**n (%)**	**n (%)**			
Understanding and participating in care (Attitudinal self-efficacy)	G1	37 (49.3)	38 (50.7)	12.8	1247	0.038^b^
	G2	17 (39.5)	26 (60.5)	13.5		
Maintaining a positive attitude (Emotional support)	G1	29 (38.7)	46 (61.3)	12.1	1360	0.150
	G2	26 (60.5)	17 (39.5)	12.9		
Seeking and obtaining information (Informational information)	G1	35 (46.7)	40 (53.3)	13.0	1454	0.363
	G2	21 (48.8)	33 (51.2)	13.4		

^a^ Adapted from Wolf et al., 2005. Possible range of score for each subscale is 4 to 16; higher score indicates higher self-efficacy

^b^ Statistically significant findings

Table 7 presents the coefficients (β), standard error, and *p*-value of the multiple regression model of the number of counseling sessions, the socio-demographic characteristics, the variables of the care pathway, the number of themes addressed, and the CASE-cancer score. The results show that G2 patients have, on average, a CASE-cancer score that is 1.92 points higher than G1 patients. However, this relationship is not statistically significant (*p* = 0.280). We found that patients living with a partner and a child or in a single-parent family have a lower average score than patients who live alone (-6.9 points vs -10.17 points, respectively), and these differences are significant (*p* < 0.05). Similarly, patients who believe that they have insufficient financial resources have a lower mean score (7.93 points; *p* = 0.025) than patients who feel financially comfortable. A statistically positive relationship was also observed between the CASE-cancer score and the stage of the care pathway. Patients who were under treatment obtained a CASE-cancer score that was 5.68 points higher (*p* = 0.030) than patients who had not started treatment. Lastly, a positive relationship was found between the CASE-cancer score and the number of questions relating to the organizational themes addressed during the support (0.93 points; *p* = 0.078).

**Table 7:** Results of estimate of ordinal least squares (OLS) regression for the CASE-cancer score

CASE	Coefficient (β)	Standard error	*p*-value
**Constant**	41.57	7.96	0.000^a^
**Support (Ref: G1)**			
G2 (several support sessions)	1.92	1.74	0.280
**Age group (Ref: 25 – 34 years)**			
35 – 44 years	1.46	2.97	0.626
45 – 54 years	1.99	3.20	0.538
55 – 64 years	-1.61	3.17	0.615
65 years or older	-0.02	3.58	0.995
**Place of birth (Ref: Canada)**			
Outside Canada	1.48	2.14	0.494
**Highest level of education completed (Ref: High school diploma)**			
University studies	-1.57	2.95	0.598
College studies	2.73	2.99	0.368
**Occupation (Ref: Other)**			
Retired	4.62	3.27	0.169
Part-time worker	3.79	3.18	0.244
Full-time worker	2.39	2.62	0.371
**Composition of the household** **(Ref.: Person living alone)**			
Couple without children at home	-2.46	2.12	0.255
Couple with child/children at home	-6.90	2.38	0.008^a^
Single-parent family	-10.17	3.23	0.004^a^
Family with other persons	-2.48	4.00	0.541
**Financial situation** **(Ref.: Financially comfortable)**			
Sufficient income to meet basic needs	-3.22	1.69	0.069
Poor	-7.93	3.33	0.025^a^
**Informal caregiver for medical follow-up (Ref: Yes)**			
No	-5.02	4.22	0.245
**Time to cancer diagnosis** **(Ref: Less than one month)**			
1 to 6 months	0.52	3.50	0.883
7 to 11 months	-0.33	3.94	0.934
1 to 3 years	3.68	5.90	0.539
More than 3 years	-2.07	9.32	0.826
**Cancer** **(Ref: First diagnosis of cancer)**			
Cancer relapse	-3.60	3.47	0.308
Second or third cancer	9.69	8.64	0.272
**Metastatic cancer (Ref: Yes)**			
No	0.24	2.92	0.937
**Number of treatments received**	0.03	0.72	0.972
**Initiation of treatment** **(Ref: Less than one month)**			
1 to 6 months	-0.26	1.91	0.892
7 to 11 months	-0.32	3.88	0.935
1 to 3 years	1.63	6.02	0.789
More than 3 years	3.51	8.42	0.680
**Stage of the care pathway** **(Ref: Before treatment begins)**			
During treatment	5.68	2.48	0.030^a^
**Number of themes**			
Organizational themes	0.93	0.50	0.078^a^
Clinical themes	-0.21	0.41	0.621
Daily themes	0.69	0.60	0.260
Support theme (informational, relational, emotional)	-0.46	0.32	0.159

^a^ Statistically significant findings

### Level of psychological distress (Q5)

The patients’ score for average level of psychological distress is 6.9 points, which means that most of the patients were experiencing a low or moderate level of psychological distress. Only 12.0% of patients in group G1 and 11.6% of patients in group G2 had a high level of psychological distress, while 56% (G1) and 51.2% (G2) of patients showed a moderate level and 32% (G1) and 37.2% (G2) showed a low level. No statistically significant difference in the level of psychological distress was found between patients who had only one meeting versus those who had several.

Table 2 (columns 8 and 9) presents the average level of psychological distress score in relation to socio-demographic characteristics and the patients’ care pathway. There is only one statistically significant difference (*p* = 0.024) in the mean score for level of psychological distress in relation to the stage of the care pathway. Patients who were undergoing treatment had a lower average score than patients who had not yet started treatment at the time of the study.

Table 8 presents results from the estimation of the multiple regression model between the number of encounters, socio-demographic characteristics, variables of the care pathway, number of themes addressed, and psychological distress score. The results show that G2 patients have, on average, a higher score (0.68 points) than G1 patients, although the relationship is not statistically significant (*p* = 0.709).

**Table 8:** Ordinal least squares (OLS) regression estimation results for the score on psychological distress level

Psychological distress	Coefficient (β)	Standard error	*p*-value
Constant	14.14	8.23	0.098
**Support (Ref: G1)**			
G2 (Several support sessions)	0.68	1.80	0.709
**Age group (Ref: 25 – 34 years)**			
35 – 44 years	-0.72	3.06	0.816
45 – 54 years	-1.95	3.30	0.560
55 – 64 years	-3.04	3.27	0.362
65 years and older	-6.83	3.69	0.076
**Place of birth (Ref: Canada)**			
Outside Canada	-1.13	2.21	0.613
**Highest level of education completed** **(Ref: High school diploma)**			
University studies	-2.46	3.05	0.427
College studies	-4.48	3.09	0.158
**Occupation (Ref: Other)**			
Retired	-0.42	3.37	0.902
Part-time worker	-1.65	3.28	0.619
Full-time worker	-3.97	2.71	0.155
**Composition of the household** **(Ref: Person living alone)**			
Couple without children at home	-1.44	2.19	0.517
Couple with child/children at home	-0.42	2.46	0.865
Single-parent family	2.26	3.33	0.503
Family with other persons	-6.92	4.13	0.106
**Financial situation (Ref: Financially comfortable)**			
Sufficient income to meet basic needs	2.31	1.75	0.198
Poor	3.25	3.44	0.353
**Informal caregiver for medical follow-up (Ref: Yes)**			
No	-0.76	4.36	0.863
**Time to cancer diagnosis (Ref: Less than 1 month)**			
1 to 6 months	-0.54	1.98	0.786
7 to 11 months	2.12	4.01	0.602
1 to 3 years	3.91	6.22	0.535
More than 3 years	3.13	8.70	0.722
**Cancer (Ref: Initial diagnosis of cancer)**			
Cancer relapse	2.69	3.58	0.459
Second or third cancer	9.52	8.93	0.296
**Metastatic cancer (Ref: Yes)**			
No	0.74	3.02	0.807
**Number of treatments received**	-0.12	0.74	0.871
**Initiation of treatment (Ref: Less than one month)**			
1 to 6 months	2.62	3.61	0.474
7 to 11 months	1.16	4.07	0.779
1 to 3 years	-6.09	6.10	0.327
More than 3 years	-8.44	9.63	0.389
**Stage of the care pathway** **(Ref: Before treatment begins)**			
During treatment	-3.54	2.56	0.179
**Number of themes**			
Organizational themes	-0.40	0.52	0.455
Clinical themes	-0.21	0.43	0.627
Daily themes	-0.53	0.62	0.397
Support theme (informational, relational, emotional)	0.56	0.33	0.099

### Relationship between CASE-cancer results and level of psychological distress

The analysis found an inverse relationship between the CASE-cancer results and the level of psychological distress. Moreover, this relationship was confirmed by a more detailed analysis of changes in the proportions between the levels of psychological distress and the CASE-cancer subscales. More specifically, we observe a significantly lower proportion of participants with a severe psychological distress score who obtained a high score on the “Seeking and obtaining information” subscale (21.4%). A significantly lower proportion of participants with a moderate psychological distress level score (28.1%) had a high score on the “Maintaining a positive attitude” subscale. The same behavior is found among patients with a severe psychological distress score (7.1%) who obtained a high score on this subscale. Lastly, as can be seen in Table 9, 65.0%, 48.4% and 42.9% of the participants obtained a low, moderate, and severe psychological distress score, respectively, and a high score on the CASE-cancer “Understanding and participating in care” subscales.

**Table 9:** Relationship between CASE-cancer subscales and levels of psychological distress

CASE-cancer subscales	Level n (%)	Psychological distress n (%)			*p*-value
		**Low**	**Moderate**	**Severe**	
Seeking and obtaining information	Low ^b^	15 (37.5)	35 (54.7)	11 (78.6)	0.02512^a^
	High ^c^	25 (62.5)	29 (45.3)	3 (21.4)	
Maintaining a positive attitude	Low ^b^	13 (32.5)	46 (71.9)	13 (92.9)	< 0.0001^a^
	High ^c^	27 (67.5)	18 (28.1)	1 (7.1)	
Understanding and participating in care	Low ^b^	14 (35.0)	33 (51.6)	8 (57.1)	0.1873
	High ^c^	26 (65.0)	31 (48.4)	6 (42.9)	

^a^ Statistically significant findings

^b^ Corresponds to a score of 4 to 12

^c^ Corresponds to a score of 13 to 16

## Discussion

This study is, to our knowledge, the first to assess perceptions of the support provided by APs integrated into clinical oncology teams during the COVID-19 pandemic. It helps us understand the profiles of the people who have benefited from such support and evaluate, according to the number of encounters between APs and patients, the lived experience of patient support and its contribution. The results show that the patients who benefited from one or more encounters do not have different socio-demographic characteristics or experiences with cancer. Additionally, our analysis revealed a statistically significant difference in the mean score for level of psychological distress based on the stage of the care pathway. Specifically, patients who were in the process of receiving treatment had a lower average score compared to those who had not yet begun treatment at the time of the study.

### Type of support

In the literature, the nature of accompanying patients' support most often refers to the ability to share information, educate patients about their health problem, support them emotionally by validating that their emotions are normal, and help them navigate the health care system [25, 26]. This study has produced the same results but highlights the fact that these results are amplified if the support is extended over time. This situation was highlighted by the APs in this study who mentioned that “Sometimes, several encounters are necessary before we see patients making some progress in their journey” [27].

### Topics discussed

The topics discussed by the patients and the APs evolved, depending on whether the patients have had one encounter (G1) or several encounters (G2) with their APs. Patients who had several encounters were more likely to discuss fatigue following treatment, the repercussions of treatment on physical appearance and self-esteem, their pain and discomfort related to treatment, the emotions they experience and their management, as well as decision-making processes. They were also more likely to discuss the roles of the various health professionals, the care trajectory, and the rights of patients (e.g., refusing treatment, asking questions). In fact, during the first encounter, the discussions take place around a specific issue (e.g., a specific surgical intervention, or the start of chemotherapy and its impacts on daily life, etc.), whereas when the patients have the opportunity to meet with a AP several times, many topics are addressed, some of which are more complex, such as patient rights, and others which are more personal, such as their condition’s impact on privacy.

These results corroborate what is found in the literature [28, 29], but also what was identified by APs who were asked in focus group interviews about the topics they discussed with patients at the beginning of their involvement and 24 months later in this study [27]. They highlighted how they find it easiest to talk about these topics, particularly those concerning patients’ rights, the organization of care (trajectory, the roles of professionals and APs) and the side effects of treatments [27].

### Effect on communication and attitudinal self-efficacy

This study shows that communication and self-efficacy skills do not vary with age, place of birth, presence of a caregiver, level of education, social occupation status, the nature of the cancer, the treatment pathway, or the date of the start of treatment. It found lower scores for people with children or with financial difficulties. These results do not highly corroborate what is found in the literature on the social determinants of health, which highlight certain factors that make it possible to better cope with a health problem, including not living alone, being in a good financial situation, and holding a university degree.

The results are also surprising in relation to cancer care pathways. One would expect that patients with more experience living with cancer (recurrence of cancer; second or third cancer) would have a higher CASE score, but this is not what was found. However, our results do show better scores when the patients are in treatment as compared to before or after treatment. This may be because when they are in treatment, they are more likely to develop their communication skills and self-efficacy. In addition, by attending more than one encounter with an AP, patients are better able to understand and participate in their care.

However, the results showed that the CASE dimension most affected among patients who met with an AP more than once was “Understanding and participating in care”, confirming a result in an Australian study [30] where patients received support of a nurse specialized in breast care.

These results raise the question of whether our sample, compared to what is known of the population of people with breast cancer in Montreal, had a much higher level of education [31]. We can hypothesize here that the most economically disadvantaged patients only rarely agree to meet APs or participate in research, as found in the literature [32, 33].

### Effect on the level of psychological distress

Regarding the level of psychological distress, no difference was found between G1 and G2. However, people experienced less psychological distress during treatment than they did at the beginning or end of treatment. These results are not confirmed in other studies carried out during the COVID-19 pandemic, which report higher K6 scores [34]. Other studies show that patients suffered mainly from anxiety or stress [35-37].

First, we can hypothesize that the K6, which primarily assesses levels of psychological distress, does not properly capture the kind of anxiety experienced by the patients in our study. On the other hand, it is possible that before treatment, patients experience greater distress due to the unknowns about what lies ahead, while after treatment they are worried about returning to a more normal life and about the risk of recurrence, as has been highlighted in the literature [38, 39].

### Interaction between communication and attitudinal self-efficacy and level of psychological distress

The patients were consistent in their ability to cope with cancer and in their level of psychological distress. Our results appear to show that patients who feel capable of coping with their cancer are less likely to be in psychological distress. Also, it is possible to hypothesize that APs act simultaneously on their perceptions of the patients’ ability to cope with cancer and on their psychological state, which corroborates Bandura's work [40].

### Study limitations and suggestions for future research

Some limitations to this study should be noted. First, the study did not collect data on people who refused support or chose not to participate in the research project. Such data would have made it possible to know whether our sample was different from other patients treated at the same hospital, especially since the APs oversaw recruitment, which could have created biases.

The second limitation is the questionnaire on the level of psychological distress of (K6), which does not seem to be sensitive enough to assess the experience of people with breast cancer. We propose that future research projects use scales that will better capture anxiety and stress in people with cancer, such as the Hospital Anxiety and Depression Scale [36].

Lastly, we did not collect comparative data from people who did not receive support from an AP. A randomized pragmatic study should therefore be carried out in a real setting [41] to better capture the effect of APs in healthcare settings.

## Conclusion

In conclusion, the study provides compelling evidence that trained peer support is a viable and appreciated option for breast cancer patients. The program successfully connected over 500 patients with trained APs, with a majority of patients accepting the offer of support. This support covered a broad range of topics related to the impacts of cancer on daily life, clinical issues, and organizational aspects of care provision. Moreover, a significant proportion of patients who received support also agreed to participate in the PAROLE-Onco research project.

Importantly, this study sheds light on the potential contribution of trained peer support to patients' ability to develop self-efficacy, highlighting the benefits of ongoing accompaniment compared to one-offs. Patients appreciated having the support embedded in the clinical team, allowing them to discuss all their concerns with a resource who can listen, understand, and help them regain control over their care pathway.

This program has responded effectively to the needs and expectations of patients in a personalized way, making it possible to accompany them throughout their cancer journey. Future studies should explore the potential of this intervention with other tumor sites and include male patients. Finally, the results have set the stage for conducting pragmatic randomized or observational trial studies in order to generate conclusive data. Overall, this approach could serve as a valuable complement to existing medical care for breast cancer patients.

## Data Availability

The data used in this study is stored and anonymized. The data is not publicly available, but it may be available upon formal request (please contact the corresponding author).

## Abbreviations

AP: Accompanying patient/pair
CASE-cancer: Communication and Attitudinal Self-Efficacy scale for cancer
CHU: Centre Hospitalier Universitaire
CHUM: Centre Hospitalier Universitaire de Montréal
CISSS: Centre Intégré de Santé et de Services Sociaux
CIUSSS: Centre Intégré Universitaire de Santé et de Services Sociaux
COVID-19: Coronavirus Disease 2019
CR-CHUM: Centre de recherche du Centre Hospitalier de l’Université de Montréal
G1: Group 1
G2: Group 2
INESSS: Institut national d’excellence en santé et services sociaux
K6: Kessler Psychological Distress Scale – 6 items
OLS: Ordinal least squares
PAROLE-onco: Patient AdvisoR, an Organizational resource as a Lever for an Enhanced Oncology patient experience
PQC: Programme Québécois de Cancérologie
Qc: Québec
Q1 – 5: Questionnaires 1 to 5
REDCap: Research Electronic Data Capture
